# Silica-coated magnetic iron oxide functionalized with hydrophobic polymeric ionic liquid: a promising nanoscale sorbent for simultaneous extraction of antidiabetic drugs from human plasma prior to their quantitation by HPLC†

**DOI:** 10.1039/c8ra02109k

**Published:** 2018-08-29

**Authors:** Sahar Badragheh, Mohsen Zeeb, Mohamad Reza Talei Bavil Olyai

**Affiliations:** Department of Chemistry, Karaj Branch, Islamic Azad University Karaj Iran; Department of Applied Chemistry, Faculty of Science, Islamic Azad University, South Tehran Branch Tehran Iran zeeb.mohsen@gmail.com m_zeeb@azad.ac.ir +982133717140 +982133722831-7

## Abstract

Herein, silica-coated iron oxide nanoparticles modified with imidazolium-based polymeric ionic liquid (Fe_3_O_4_@SiO_2_@PIL) were fabricated as a sustainable sorbent for magnetic solid-phase extraction (MSPE) and simultaneous determination of trace antidiabetic drugs in human plasma by high-performance liquid chromatography-ultraviolet detection (HPLC-UV). The Fe_3_O_4_ core was functionalized by silica (SiO_2_) and vinyl layers where the ionic liquid 1-vinyl-3-octylimidazolium bromide (VOIM-Br) was attached through a free radical copolymerization process. In order to achieve hydrophobic magnetic nanoparticles and increase the merits of the sorbent, Br^−^ anions were synthetically replaced with PF_6_^−^. The properties and morphology of the sorbent were characterized by various techniques and all the results illustrated the prosperous synthesis of Fe_3_O_4_@SiO_2_@PIL. A comprehensive study was carried out to investigate and optimize various parameters affecting the extraction efficiency. The limit of detection (LOD, S/N = 3) for empagliflozin, metformin and canagliflozin was 1.3, 6.0 and 0.8 ng mL^−1^, respectively. Linearity (0.997 ≥ *r*^2^ ≥ 0.993) and linear concentration ranges of 5.0–1200.0, 20.0–1800.0 and 5.0–1000.0 ng mL^−1^ were obtained for empagliflozin, metformin and canagliflozin, respectively. Intra-assay (3.8–7.5%, *n* = 9) and inter-assay (3.2–8.5%, *n* = 12) precisions as well as accuracies (≤9.1%) displayed good efficiency of the method. Finally, the method was applied for the quantitation of antidiabetic drugs in human plasma after oral administration and main pharmacokinetic data including *T*_max_ (h), *C*_max_ (ng mL^−1^), AUC_0–24_ (ng h mL^−1^), AUC_0–∞_ (ng h mL^−1^), and *T*_1/2_ (h) were evaluated.

## Abstract 2

A sustainable nanoscale core–shell modified with hydrophobic polymeric ionic liquid was fabricated for simultaneous extraction and determination of antidiabetic drugs.

## Graphical abstract

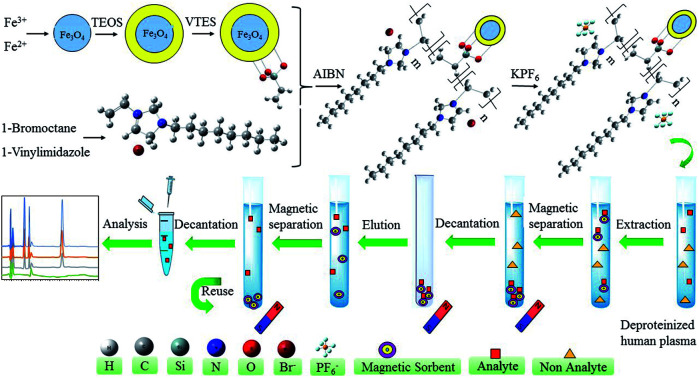

## 1. Introduction

**Fig. 1:**
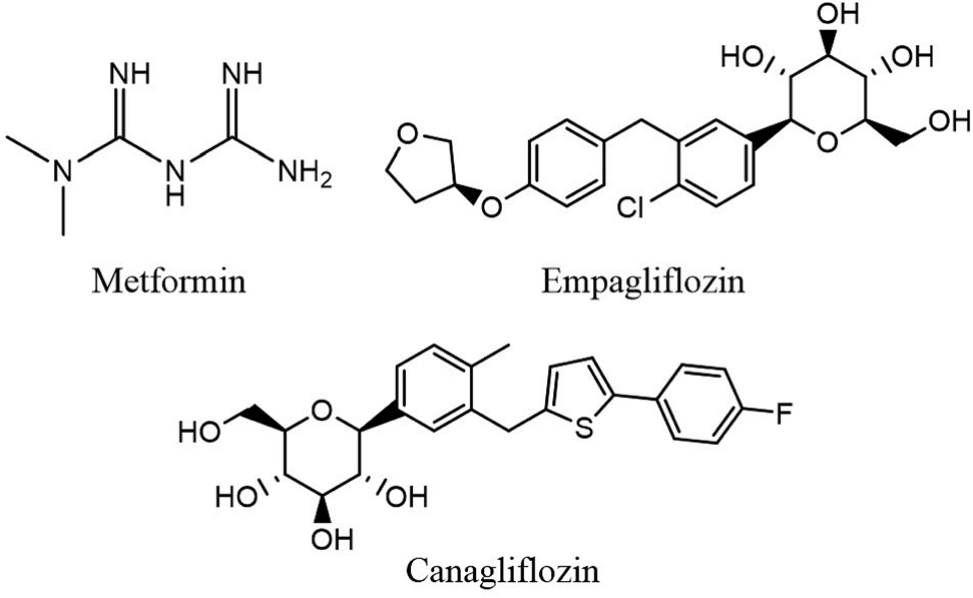
Chemical structures of EMPA, MET, CANA.

Type 2 diabetes mellitus (T2DM) and its abnormalities constitute a major metabolic disease affecting human health seriously in modern society.^1^ T2DM is a heterogeneous group of disorders characterized by abnormalities in carbohydrate, protein, and lipid metabolism which can cause serious health complications including ketoacidosis, kidney failure, heart disease, stroke and metabolic syndrome (dyslipidemia, hypertension). Furthermore, for many patients with T2DM, monotherapy with an oral antidiabetic agent is not adequate and multiple drugs may be necessary to achieve glycemic control in the long term. Thus, a combination formulation which includes drugs with different and complementary mechanisms of action would potentially offer increased terms of convenience and patient compliance.^2–4^ Therapeutic drug monitoring is essential to ensure the measurement of plasma concentration for pharmacokinetic studies, bioequivalence assessment of commercially available tablet formulation, optimization of novel dosage forms and dosing regimen in combination therapy for diagnostic purposes.^5,6^ Thus, development of reliable, sensitive and rapid analytical methods is required to simultaneously determine trace amounts of antidiabetic drugs in human plasma. The structures of three antidiabetic drugs chosen for this study including metformin (MET), empagliflozin (EMPA) and canagliflozin (CANA) are shown in Fig. 1.

Due to the complex matrix of plasma, sample preparation methods are often required prior to chromatographic analysis. There are several techniques described in the literature for extraction of drugs in biological matrices such as liquid–liquid extraction (LLE), dispersive liquid–liquid microextraction (DLLME), solid phase extraction (SPE), micro-solid-phase extraction (μ-SPE), solid-phase membrane micro-tip extraction (SPMMTE), *etc.*^7–10^ In this case, Saha and coauthors have described probe electrospray ionization mass spectrometry to detection of drugs from plasma samples with no or minimal sample pre-treatment.^11^ Recent attempts in sample preparation are particularly focused on improving the quality of analytical results and introducing new technological expansions such as simplification, miniaturization and automation of the whole analytical method.

Due to some notable properties of iron-oxide nanoparticles including the intrinsic magnetism, low toxicity, facile preparation and high surface area-to-volume ratio, they are the most frequently used sorbents in MSPE.^12–15^ So far, various kinds of surface modifier such as carbon nanotubes (CNTs),^16^ graphene,^17^ polymer^18^ and cyclodextrins^19^ have been utilized as exterior shell material to adjust the properties of sorbents. Core–shell structure is a common existing form for MSPE sorbents, and lately it has received great attention for different screening purposes.^20^ Among these magnetic hybrid materials, modifying Fe_3_O_4_ with ionic liquids (ILs) on the shell moieties has been attracting growing attention.^21,22^

Beyond the excellent physicochemical and thermal properties of the ILs, they are also recurrently recognized by their excellent solvation ability for a wide range of compounds.^23–26^ Lately, ILs have been extremely used as green extraction media in some enrichment methods for different monitoring purposes.^27^ In our previous works, ILs were successfully used in micro-scale extraction methods to determine trace levels of atenolol and zinc in human plasma and food samples, respectively.^28,29^ Physically immobilization of ILs on the surface of Fe_3_O_4_ particles can merge the advantages of ILs and magnetic materials which resolves this serious drawback of ILs. Chen and his coworkers indicated that physically modification of Fe_3_O_4_@SiO_2_ particles with [OMIM]PF_6_ could provide practical sorbent for extraction of bisphenol A.^30^ However, physically adsorption of ILs on the surface of nanoparticles is truly unstable and results in poor reusability.^31–33^ For this presently reason, ILs have been covalently bonded to magnetite to significantly improve the stability and minimize ILs loss during extraction procedure.^34^

Literature survey revealed that polymeric bonds offer strong adhesion between the IL coating and the magnetic nanoparticles (MNPs), which prevents ionic liquids leakage.^35–38^

In this study, silica-coated iron oxide nanoparticles were functionalized with PIL (poly-1-vinyl-3-octylimidazolium bromide (VOIM-Br)) by a free radical copolymerization process in order to fabricate an efficient magnetic recyclable sorbent. Br^−^ anions of PIL were synthetically changed with PF_6_^−^ anions to increase hydrophobic character of the designed solid phase. The new sorbent possesses the advantages of both PILs and the magnetic iron oxide. Three antidiabetic drugs involving EMPA, MET and CANA were selected as the model compounds to ascertain the feasibility of this sorbent for simultaneous extraction and preconcentration prior to quantification by HPLC-UV. To the best of our knowledge, this work is the first report on the application of Fe_3_O_4_@SiO_2_@PIL-PF_6_ for trace determination of antidiabetic drugs in human plasma. The predominant factors affecting the extraction efficiency of the analytes were evaluated in detail and optimized with one at a time approach. Ultimately, the optimum conditions were successfully used to investigate the applicability of the proposed method for the determination of antidiabetic drugs in 8 healthy fed participants and main pharmacokinetic data were achieved.

## 2. Experimental

### 2.1. Chemicals

1-Vinylimidazole and potassium hexafluorophosphate (KPF_6_) were purchased from Sigma-Aldrich (St. Louis, MO, USA). Iron(iii) chloride hexahydrate (FeCl_3_·6H_2_O), iron(ii) chloride tetrahydrate (FeCl_2_·4H_2_O), ammonia, tetraethyl orthosilicate (TEOS), 1-vinyltriethoxysilane (VTES), 2,2′-azobisisobutyronitrile (AIBN), 1-bromooctane and triethylamine were obtained from Merck Chemicals (Darmstadt, Germany). HPLC grades of methanol, acetonitrile, acetone, potassium dihydrogen phosphate and sodium dodecyl sulfate were acquired from Merck (Darmstadt, Germany). Standards of CANA and EMPA were supplied from MSN Life Science Private Limited Unit-2, (India) and Emeishan Hongsheng Pharmaceutical Co. (China), respectively. Standard of MET kindly donated by Mahban Chemi Co. (Tehran, Iran). Fixed-dose combination Invokamet tablets (50 mg CANA/500 mg MET) and Jardiance tablets (25 mg EMPA) were purchased from Janssen (USA). Fresh plasma samples were obtained from Iranian Blood Transfusion Organization (Tehran, Iran) and stored at −18 °C until being used. Ultrapure water (Millipore, Bedford, MA, USA) was used throughout the whole experiments.

### 2.2. Apparatus

Fourier transform infrared (FT-IR) spectral studies were carried out by a Vector 22 FT-IR spectrometer (Bruker, Germany) over the wavenumber range of 400–4000 cm^−1^. The ^1^H NMR spectra of synthesized IL were recorded with a Avance DRX-500 MHz spectrometer instrument (Bruker, Germany). X-ray diffraction (XRD) patterns were obtained with a D8 Advance X-ray diffractometer (Bruker, Germany) using Cu Kα radiation source (*λ* = 1.54059 Å). Thermal gravimetric analysis (TGA) were carried out using a Pyris 1 TGA instruments (Perkin Elmer, USA) operated with a heating rate of 15 °C min^−1^ from 25 °C to 800 °C under oxygen atmosphere. The size and morphology of the as-synthesized nanoparticles were studied using a SIGMA VP-500 field emission scanning electron microscope (FESEM) (Zeiss, Germany) and a Tecni F20 transmission electron microscopy (FEI, Hillsboro, OR, US). The elemental composition was characterized by an energy dispersive X-ray fluorescence spectroscopy (EDXRF) (Oxford Instrument, UK) attached to the Zeiss FESEM. Magnetization measurement was performed using a vibrating sample magnetometer (VSM/AGFM Meghnatis Daghigh Kavir Co., Kashan) at room temperature by cycling the field from −10 to 10 kOe. A Sonorex ultrasonic water bath (Misonix-S3000, USA) equipped with a digital timer and a temperature controller, a WTW Inolab pH meter (Germany), a CL centrifuge (International Equipment Co, USA) and a Heidolph heating magnetic stirrer (Schwabach, Germany) were used.

### 2.3. HPLC condition

The chromatographic analysis was conducted using a Waters alliance e2695, (Massachusetts, USA) system which was equipped with a Waters 2487 dual wavelength detector and C_18_ TMS endcapping/reversed phase column (Luna 5 μm C_18_ 100 A HPLC column 250 × 4.6 mm id, Phenomenex Co, Torrance, CA) at 40 °C. The mobile phase which being used in an isocratic mode was composed of potassium dihydrogen phosphate and sodium dodecyl sulfate (0.01 M, pH 6): acetonitrile (55 : 45, v/v) and passed through column with flow rate of 1 mL min^−1^ (the mobile phase was filtered and degassed daily prior to use in HPLC). In the case of metformin, the retention time of this drug was too short and overlapped with solvent peak which was due to its charge. In order to retain this drug in the column and obtain better separation condition a surfactant like dodecyl sulfate was used as an ion-pairing agent. The column effluent was monitored at UV wavelength of 215 nm and the injection volume was 20.0 μL. The mobile phase was filtered by passing it through a 0.2 μm membrane filter (Millipore, Bedford, MA, USA).

### 2.4. Synthesis of hydrophobic Fe_3_O_4_@SiO_2_@PIL-PF_6_ MNPs

The synthesis of the Fe_3_O_4_@SiO_2_@PIL-PF_6_ was carried out following six well-defined steps, which are explained below:

#### 2.4.1. Synthesis of 1-vinyl-3-octylimidazolium bromide monomers, (VOIM-Br)

1-Vinyl-3-octylimidazolium bromide monomers was synthesized by direct reaction of raw materials following the modified procedure described elsewhere.^39^ 1-vinylimidazole (0.05 mol) and 1-bromooctane (0.055 mol, 10% excess) was heated and maintained at 60 °C for 4 h and agitated with a magnetic stirrer. After cooling down to room temperature, obtained brownish-yellow viscous ionic liquid monomer was extracted with toluene several times to remove the remaining starting materials. Due to the high viscosity of IL monomers, in each washing step, the solution was allowed to stand and the supernatant liquid was discarded. Finally, the resulting IL monomers was dried under vacuum.

#### 2.4.2. Synthesis of iron oxide magnetic nanoparticles, Fe_3_O_4_

In order to synthesis magnetic Fe_3_O_4_ nanoparticles, FeCl_3_·6H_2_O and FeCl_2_·4H_2_O with the molar ratio of 2 : 1 (FeCl_3_·6H_2_O (10.8 g) and FeCl_2_·4H_2_O (4.0 g)) were dissolved in 250.0 mL of ultrapure water. The solution was vigorously stirred at 80 °C for 30 min. Later on, 20.0 mL of ammonia solution (25% (w/w)) was added dropwise into the mixture. The resultant solution was vigorously stirred at 80 °C for 60 min and N_2_ was used as the protective gas in the whole experiment. After completion of the reaction and cooling to room temperature, the obtained black precipitate was separated from the reaction media using an external magnetic field. Next, it was washed with water and ethanol in order to remove the unreacted chemicals. Finally, the resulting material was dried in vacuum.

#### 2.4.3. Synthesis of silica-coated iron oxide magnetic nanoparticles, Fe_3_O_4_@SiO_2_ MNPs

For this purpose, previously reported biphase method was applied with some modifications.^40^ The silica coated core–shell magnetic nanoparticles (Fe_3_O_4_@SiO_2_MNPs) were prepared by an ultrasonic premixing of the black precipitate of Fe_3_O_4_ (2.0 g) with deionized water (400.0 mL) for approximately 10 min at room temperature. Then, the pH value of the solution was adjusted to 9–11 using aqueous NH_3_ (25% (w/w)) and afterwards 16.0 mL TEOS were slowly added. The resulting solution were refluxed at 90 °C under nitrogen protection, during continuous stirring for 2 h. After cooling to ambient temperature the black precipitate product (Fe_3_O_4_@SiO_2_) was collected by magnetic separation and rinsed with water and ethanol thoroughly before being dried under vacuum.

In this step, according to our scientific experience, the chemical stability of Fe_3_O_4_ and Fe_3_O_4_@SiO_2_ MNPs were analyzed *via* comparative acid corrosion experiment to be certain about the successful silica coating of iron nanoparticles. For Fe_3_O_4_ the black color of the solution was changed upon the addition of concentrated HCl (turned to yellow) and mixture limpid immediately but no significant change occurred for Fe_3_O_4_@SiO_2_ (Fig. S1†). This observation confirms triumphant coating of Fe_3_O_4_ and resistance enhancement of the fabricated core against oxidation and corrosion using TEOS.

#### 2.4.4. Synthesis of vinyl modified silica-coated iron oxide magnetic nanoparticles, Fe_3_O_4_@SiO_2_@VTES MNPs

In order to prepare Fe_3_O_4_@SiO_2_@VTES, 0.5 g Fe_3_O_4_@SiO_2_ was dispersed in dry distilled toluene (40.0 mL) with the aid of ultrasonication for 10 min. Next, 0.3 mL triethylamine (as a catalyst) and VTES were added. The mixture was refluxed in an oil bath during 12 h with magnetic stirring under nitrogen stream. After naturally cooling to room temperature, the resulting material was gathered by magnetic separation, washed several times with toluene and methanol to eliminate excess reactants and dried under vacuum.

#### 2.4.5. Synthesis of silica-coated iron oxide magnetic nanoparticles functionalized with polymeric ionic liquid, Fe_3_O_4_@SiO_2_@PIL-Br MNPs

PIL was immobilized on the magnetic nanoparticle surface by the free radical copolymerization using AIBN as an initiator through a simple reflux. Freshly prepared Fe_3_O_4_@SiO_2_@VTES nanoparticles (0.5 g) were suspended in 2-propanol and the resulted suspension was sonicated for 10 min in ultrasonic bath to gain a homogeneous solution. It was followed by addition of AIBN and 1-vinyl-3-octylimidazolium bromide monomers through stirred continuously for 4 h while nitrogen gas was purged. The resultant product was permitted to cool, isolated by magnet, washed sequentially with distilled water and then dried in vacuum.

#### 2.4.6. Synthesis of silica-coated iron oxide magnetic nanoparticles functionalized with hydrophobic polymeric ionic liquid, Fe_3_O_4_@SiO_2_@PIL-PF_6_ MNPs

**Fig. 2:**
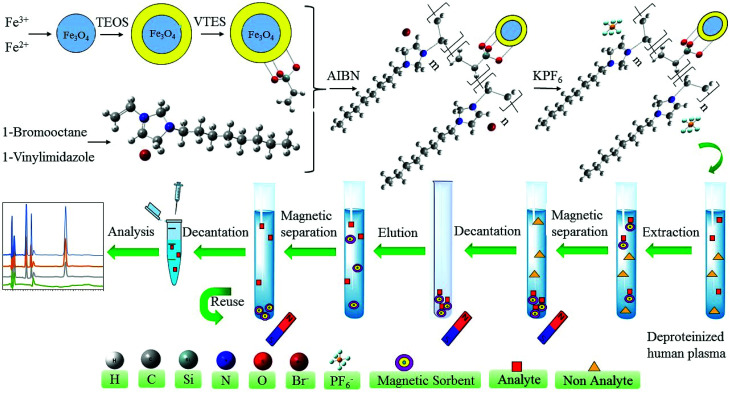
Schematic diagram of the preparation Fe_3_O_4_@SiO_2_@PIL MNPs and its application as MSPE sorbent for extraction and preconcentration of target drugs.

The last step of the synthesis is the substitution of the Br^−^ by PF_6_^−^ anions on the polymeric ionic liquid modified magnetic nanoparticles to obtain hydrophobic sorbent. Briefly, 2.0 g of Fe_3_O_4_@SiO_2_@PIL-Br MNPs were added in the deionized water and ultrasonically dispersed for 10 min. After dispersion, 1.0 g of KPF_6_ was dissolved in water and then added gradually into a beaker which contains Fe_3_O_4_@SiO_2_@PIL-Br MNPs. Solution stirred vigorously at room temperature overnight. Subsequently, MNPs were gotten by magnetic separation, washed with water and methanol and dried at room temperature. The solid was carefully grinded into powders and used for further studies. The graphical synthesis route was depicted in Fig. 2.

### 2.5. Preparation of standard solutions and quality control samples

Stock standard solutions of CANA, EMPA and MET (100.0 mg L^−1^) were prepared separately by dissolving proper amounts of each drug in HPLC grade methanol. These solutions were substituted every week with new ones to prevent decomposition of the drugs. The working standard solutions were daily prepared by appropriate stepwise dilution of the stock standard solutions with deionized water to the desired concentration. Standards for plotting calibration curve were provided by spiking the working solutions into human plasma. Quality control (QC) samples of each drug at concentration levels of 25.0, 500.0 and 1000.0 ng mL^−1^ were prepared for evaluating the accuracy and precision of the method. All the solutions were stored in a dark brown glass vials under refrigeration at −18 °C and brought to ambient temperature just prior to use.

### 2.6. Plasma sample preparation

Every frozen human plasma were firstly thawed at room temperature and placed into a sample glass tube. Preparation of plasma before MSPE method was based on protein precipitation with aid of acetonitrile. In this regard, the plasma (1.9 mL) was fortified with 100.0 μL of required concentrations of analytes followed by mixing with 2.0 mL ACN. The obtained solution was vortexed for 1 min and centrifuged for 5 min at 3500 rpm. Then, ACN content of the clear supernatant was evaporated under nitrogen stream and the remaining sample was transferred into 4.0 mL buffer solution at pH 4.0. Sample volume after evaporation under nitrogen stream was about 2 mL and total sample volume after transferring to 4 mL buffer solution was about 6 mL. 5.0 mL of the deproteinized samples containing target drugs were subjected to the extraction procedure for subsequent analysis.

### 2.7. Recommended MSPE procedure

The experimental MSPE setup is shown in Fig. 2. Initially, 5.0 mL of the deproteinized human plasma (initial volume of spiked plasma sample 2.0 mL) was placed into a glass tube and 10.0 mg of magnetic Fe_3_O_4_@SiO_2_@PIL was added to the sample. The mixture was sonicated for 4 min to totally disperse the magnetic sorbent through the matrix and complete the extraction of target drugs at room temperature. Analyte-loaded sorbent was collected from the sample solution by exposing the glass tube to a powerful neodymium–iron–boron (Nd–Fe–B) magnet (5 × 5 × 4 cm, 0.8 tesla), and the upper phase was poured out. Later, the adsorbed analytes were eluted by 1.0 mL of acetonitrile (0.5 mL every time and washed two times) to desorb the analytes. Elution process was completed during 2 min in the ultrasonic bath. The magnet was used again to separate the sorbent and then the total volume of eluted solution was collected and evaporated to dryness under stream of the nitrogen. The dry residue was re-dissolved in 100.0 μL of mobile phase and stirred for 1 min. Finally, 20.0 μL of this solution was injected into the HPLC-UV system for analysis.

## 3. Results and discussion

### 3.1. Characterization of the Fe_3_O_4_@SiO_2_@PIL

#### 3.1.1. Proton nuclear magnetic resonance (^1^HNMR)

^1^H NMR spectrum of ionic liquid monomer is given in ESI (Fig. S2†) and the data are as follows: ^1^H NMR (DMSO-*d*_6_, ppm, 500 MHz): *δ* 0.82 (t, 3H), 1.23 (m, 10H), 1.81 (qi, 2H), 4.21 (t, 2H), 5.40 (dd, 1H), 6.00 (dd, 1H), 7.34 (dd, 1H), 8.00 (d, 1H), 8.28 (d, 1H), 9.74 (s, 1H).

#### 3.1.2. Transmission electron microscopy (TEM)

**Fig. 3:**
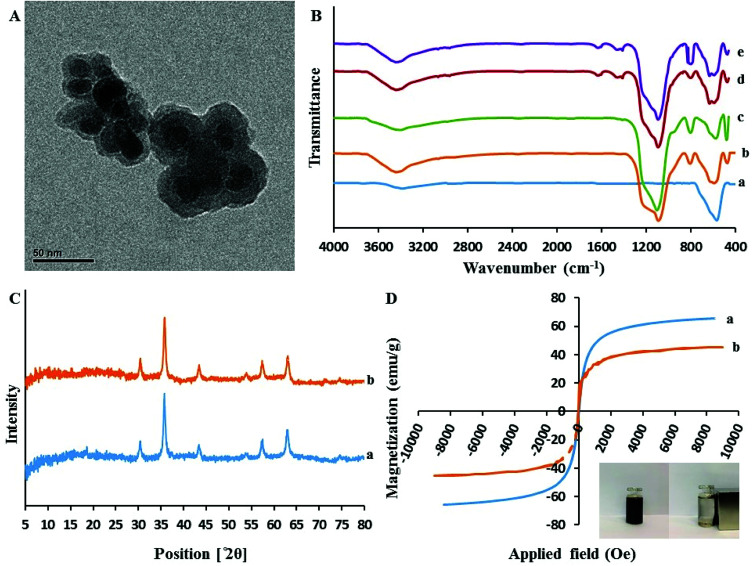
(A) The transmission electron microscopy image of Fe_3_O_4_@SiO_2_@PIL-PF_6_; (B) The FT-IR spectra of Fe_3_O_4_ (a), Fe_3_O_4_@SiO_2_ (b), Fe_3_O_4_@SiO_2_@VTES (c). Fe_3_O_4_@SiO_2_@PIL-Br (d) Fe3O4@SiO_2_@PIL-PF_6_ (e); (C) XRD pattern of the Fe_3_O_4_ (a) and Fe_3_O_4_@SiO_2_@PIL-PF_6_ (b) nanoparticles; (D) Magnetic hysteresis loops of Fe_3_O_4_ (a) and Fe_3_O_4_@SiO_2_@PIL-PF_6_ (b).

As presented in Fig. 3A, the core–shell structure of sorbent can be clearly distinguished. In fact, the Fe_3_O_4_ cores are surrounded by a dense layer of SiO_2_@PIL-PF_6_ shell with the mean thickness of approximately 10 nm (the Fe_3_O_4_ core is black while the SiO_2_@PIL-PF_6_ shell is grey). Furthermore, this image depict that the particles are rather monodisperse and have uniform spherical shape with homogeneous distribution. They have average particle size of 15–20 nm and no free nanoparticles can be found.

#### 3.1.3. Fourier transform infrared spectroscopy (FT-IR)

Fig. 3B(a–d) represents the comparative FTIR spectra of Fe_3_O_4_ (a), Fe_3_O_4_@SiO_2_ (b), Fe_3_O_4_@SiO_2_@VTES (c), Fe_3_O_4_@SiO_2_@PIL-Br (d) in the range of 4000–400 cm^−1^. All the spectra exhibit a clear and well distinct band at 570 cm^−1^ that can be assigned to the Fe–O–Fe stretching vibration. It is obvious that after coating of Fe_3_O_4_ with SiO_2_, vinyl and then polymerization with IL, the signal of Fe–O stretching peak is decreased. The presence of sharp and strong peaks at 1085 cm^−1^ corresponds to Si–O–Si and Si–O–H stretching vibrations and broad peaks located in region of 3200–3675 cm^−1^ could be assigned to the hydroxyl groups stretching vibration attached by the hydrogen bonds to the iron oxide surface. In comparison with Fe_3_O_4_@SiO_2_ (Fig. 3B(b)), the absorption band of Fe_3_O_4_@SiO_2_@VTES (Fig. 3B(c)) at 3200–3675 cm^−1^ was reduced which revealing that most hydroxyl groups were reacted with ethoxy groups of VTES and also implying to successful coating with VTES. As it is clear in Fig. 3B(d), additional new peaks at 1636, 2958 and 3092 cm^−1^ were attributed to the stretching vibration of imidazolium ring and methylene units, respectively. These results reveal the successful immobilization of PIL onto the surface of the magnetic nanoparticles.

#### 3.1.4. X-Ray diffraction (XRD)

The XRD patterns of these materials are illustrated in Fig. 3C. In the 2*θ* range of 5°–80°, six characteristic X-ray diffraction peaks at 30.3° (220), 35.7° (311), 43.3° (400), 53.7° (422), 57.3° (511) and 62.9° (440) were observed in both XRD patterns of Fe_3_O_4_ (a) and Fe_3_O_4_@SiO_2_@PIL-PF_6_ MNPs (b). These results are in good agreement with the Joint Committee on Powder Diffraction Standards (JCPDS card) of Fe_3_O_4_ and indicated a cubic spinel structure of the magnetite. According to this pattern we can conclude that the magnetic crystalline profile of the resultant Fe_3_O_4_@SiO_2_@PIL-PF_6_ remains unchanged during the modification process. Average diameters of as-synthesized Fe_3_O_4_ and Fe_3_O_4_@SiO_2_@PIL-PF_6_ MNPs based on the reflection peak of 311 (2*θ* = 35.7) were calculated using the Debye–Scherer equation (*D* = *Kλ*/*β* cos *θ*) and they found to be 18.15 and 17.78 nm, respectively which is in agreement with TEM results.

#### 3.1.5. Vibrating sample magnetometer (VSM)

In Fig. 3D(a and b) the magnetic hysteresis loops of Fe_3_O_4_ (a) and Fe_3_O_4_@SiO_2_@PIL-PF_6_ (b) are presented. The two S-like shape of magnetization curve confirm that these two compounds are super-paramagnetic which is due to the core magnetite particles. The maximum saturation magnetization of Fe_3_O_4_ (a) and Fe_3_O_4_@SiO_2_@PIL-PF_6_ (b) were 65.65 and 45.45 emu g^−1^, respectively. As shown in digital photographs in Fig. 3D (inset), all the Fe_3_O_4_@SiO_2_@PIL-PF_6_ MNPs could be separated from the solution with the aid of strong external magnet.

#### 3.1.6. Thermogravimetric analysis (TGA)

TGA was conducted in an oxygen atmosphere over a temperature range of 25–800 °C and the heating rate of 15 °C min^−1^ for Fe_3_O_4_@SiO_2_@VTES and Fe_3_O_4_@SiO_2_@PIL-PF_6_. From the experimental results which are shown in Fig. S3(a and b)† it can be concluded that: (1) weight loss of approximately 1.00% below 160 °C corresponds to loss of the adsorbed water in both materials, (2) an increase in the Fe_3_O_4_@SiO_2_@VTES curve (a) is related to the oxidation of vinyl groups and (3) a second mass loss about 3.5% of Fe_3_O_4_@SiO_2_@PIL-PF_6_ in the range of 200–550 °C is attributed to the thermal decomposition of the PILs (b).

#### 3.1.7. Field emission scanning electron microscopy (FESEM)

**Fig. 4:**
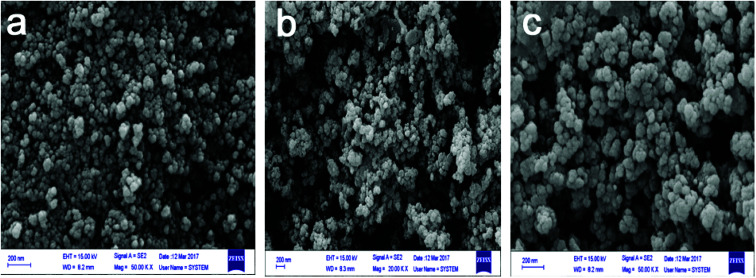
The field emission scanning electron microscopy images of Fe_3_O_4_ (a), Fe_3_O_4_@SiO_2_ (b) and Fe_3_O_4_@SiO_2_@PIL-PF_6_ (c).

FESEM images of Fe_3_O_4_, Fe_3_O_4_@SiO_2_ and Fe_3_O_4_@SiO_2_@PIL(PF_6_) were recorded and exhibited in Fig. 4(a–c). At first glance, the round surface of MNPs is obvious which provides superior active surfaces area for the adsorption of the target drugs during MSPE. Rougher structure of Fe_3_O_4_@SiO_2_@PIL-PF_6_ (c) than Fe_3_O_4_ (a) could be attributed to successful surface coating. Also it can be evidently concluded from FESEM images, the size of magnetic nanoparticles after anchoring the PIL onto the silica-coated iron oxide is not significantly changed and is still in dimension of nanometers. This exploration reveals that Fe_3_O_4_@SiO_2_@PIL-PF_6_ MNPs were coated by a thin layer of the polymeric ionic liquid providing abundant various active reaction sites for grafting.

#### 3.1.8. Energy dispersive X-ray fluorescence spectroscopy (EDXRF)

The elemental composition of the nano-sized sorbent was also determined using an energy dispersive X-ray fluorescence spectroscopy (EDXRF) system coupled with FESEM. The EDXRF spectra are shown in Fig. S4(a–c).† EDXRF spectrum of Fe_3_O_4_ clearly specifies the presence of iron (Fe) and oxygen (O) elements (Fig. S4a†). Meanwhile, following the modification process the results unambiguously corroborates the existence of iron (Fe), oxygen (O), silicon (Si) in Fe_3_O_4_@SiO_2_ (Fig. S4b†), and iron (Fe), oxygen (O), silicon (Si), carbon (C), nitrogen (N), phosphorus (P) and fluorine (F) in Fe_3_O_4_@SiO_2_@PIL-PF_6_ (Fig. S4c†). In order to access elemental distribution of the sorbent, elemental mapping was studied (Fig. S5†).

### 3.2. Optimization of extraction conditions

#### 3.2.1. Effect of Fe_3_O_4_@SiO_2_@PIL-PF_6_ MNPs amount

**Fig. 5:**
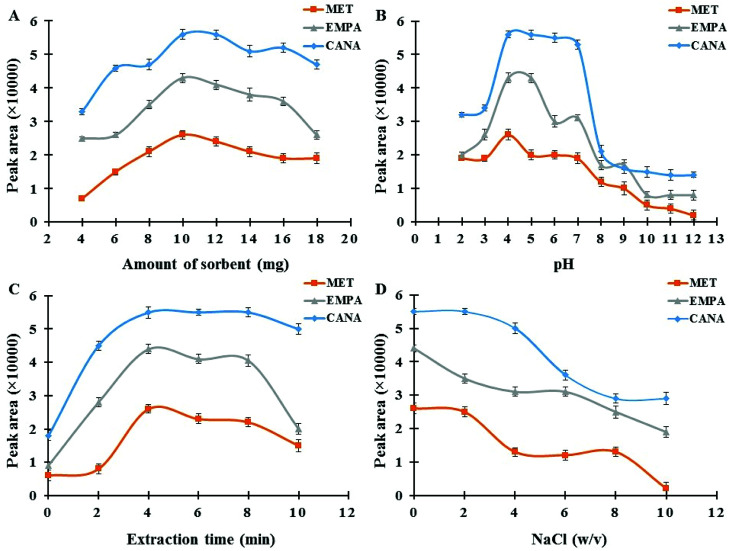
(A) Effect of Fe_3_O_4_@SiO_2_@PIL-PF_6_ MNPs amount; experimental conditions: concentration of each drug 250.0 ng mL^−1^; pH 4.0; extraction time 4 min; desorption solvent ACN; desorption time 2 min. (B) Effect of sample pH; experimental conditions: concentration of each drug 250.0 ng mL^−1^; sorbent amount 10.0 mg; extraction time 4 min; desorption solvent ACN; desorption time 2 min. (C) Effect of extraction time; experimental conditions: concentration of each drug 250.0 ng mL^−1^; sorbent amount 10.0 mg; pH 4.0; desorption solvent ACN; desorption time 2 min. (D) Effect of ionic strength; experimental conditions: concentration of each drug 250.0 ng mL^−1^; sorbent amount 10.0 mg; pH 4.0; extraction time 4 min; desorption solvent ACN; desorption time 2 min.

The dosage of the magnetic sorbent seems to be an important practical parameter, which affects the extraction efficiency and subsequent measurements.^41^ The required amount of MNPs for the complete separation and recovery of antidiabetic drugs (concentration level of 250 ng mL^−1^) was studied in the range of 2–18 mg. As it can be seen in Fig. 5A, maximum extraction efficiency was achieved at 10.0 mg of MNPs. These satisfactory results reveal that quantitative recoveries can be obtained with low amounts of this nano-sized sorbent in comparison with ordinary micro-sized sorbents in SPE, which is due to the advantage of high surface area in NPs.^42^ Metformin has no aromatic ring whereas empagliflozin, and canagliflozin have aromatic rings and more resonance systems which this fact affects on the absorbance coefficient in UV region, the kind and efficiency of interactions between sorbent and drugs (for example π–π interactions) and subsequent extraction effectiveness. However, by increasing the dosage from 10.0 mg the extraction efficiency of the target analytes decreases slightly. This might be attributed to the fact that all magnetic nanoparticles may not be separated effectively and were suspended in the solution at the same duration. Furthermore, the accessible surface of the sorbent for sorbent–drug interactions reduces owing to the aggregation of the adsorbent in higher amounts which finally leads to a decrease in extraction efficiency. Hence, 10.0 mg of sorbent was selected as the optimum and applied for the rest of the experiments.

#### 3.2.2. Effect of sample pH

The pH of sample media is one of the most important factors controlling the adsorption performance of the analytes on Fe_3_O_4_@SiO_2_@PIL MNPs.^43^ In order to achieve the best extraction condition, the impact of sample pH was evaluated within the range of 2.0–12.0 using 0.01 M HCl and NaOH. The results in Fig. 5B indicated that maximum adsorption performance and reproducible data occurred at pH 4.0. Moreover, MNPs exhibit no adsorption improvement at pH values above 4. MET has acid dissociation constant values (p*K*_a_) of 2.8 and 11.5 and, thus at pH values around 7 like physiological media it exists as the hydrophilic cationic species.^44^ But by changing the pH solution the molar ratio of uncharged form to charged form of metformin alters which affects on its solubility in water and hydrophobic property. Recently, Desai and co-workers have reported that metformin hydrochloride tablets dissolved more slowly in pH 1.2 and 4.5 as compared with pH 6.8.^45^ They have reported that at pH range of 1.2 to 4.5 cationic metformin forms an insoluble crystalline salt through an interaction with anionic compounds (as an ion-paring agent) like surfactants, but this phenomenon does not take place at pH 6.8. So, at pH around 7 partition coefficient of MET in biphasic system of *n*-octanol and water is low which is due to the positive charge of MET. But in real samples like human plasma or tablets due to the presence of many anionic compounds, the conditions may be different as Desai and co-workers reported. These results reported in literature are in agreement with the data obtained in the recent study and it seems at pH 4.0 insoluble crystalline of MET (as hydrophobic species) interacts with hydrophobic sorbent leading to the desirable adsorption of the drug. Also, CANA and EMPA with alcoholic groups are weak acids and therefore, at low pH values uncharged forms of analytes are predominate and thus stronger hydrophobic–hydrophobic interactions take place resulting better extraction performance. Hence, pH 4.0 was selected as the optimum for the following studies.

#### 3.2.3. Effect of extraction time

Adequate ultrasound time assurances the complete dispersion of the sorbent through the sample media and provides better mass transfer and higher recovery values.^46^ The influence of this variable was investigated in the range of 0–10 min. As it can be seen in Fig. 5C(a) duration time of 4 min was suitable to reach satisfactory extraction equilibrium. When the extraction time was more than 4 min, recoveries of the analytes decreased slightly. This observed drop in signals probably indicates that with an increase in the extraction time some parts of the analytes return to the solution and resulting a minor decrease in recovery. Accordingly, an extraction time of 4 min was selected for the rest of the work.

#### 3.2.4. Effect of desorption condition

Three organic solvents involving methanol, acetonitrile and acetone were selected for eluting the analytes. The analytical signals obtained for the target drugs indicated that the desorption ability of ACN was much stronger than the other organic media. So, ACN was chosen as the optimum desorption agent in all experiments. To investigate the effect of eluent volume on the desorption efficiency, volumes ranging from 0.5–4.0 mL were tested. The experimental results revealed that with increasing the amount of ACN up to 1.0 mL extraction efficiency increased. This was due to the good dispersion of the sorbent in this medium, which provided more interaction between the sorbent and drugs. Further increase in the solvent volume has no significant consequence on the extraction efficiency. Hence, 1.0 mL of desorption solvent was utilized as the optimum volume for the next studies to ensure efficient elution of the analytes. However, using 0.5 mL in two times washing offered more steady analytical responses. The influence of desorption time on the extraction performance was studied from 0.5 to 5 min and the results indicated that 2 min was sufficient enough to elute and desorb the drugs from the MNPs.

#### 3.2.5. Effect of ionic strength

Due to salting out effect, addition a salt like NaCl causes a reasonable increase in ionic strength of sample media resulting an improvement in extraction performance.^47^ The influence of ionic strength on the extraction efficiency of antidiabetic drugs was also explored by adding various amounts of sodium chloride over the concentration range of 0–10% w/v in the sample solution. According to the results provided in Fig. 5D, by raising the salt concentration a notable reduction in signals was observed. It was due to the fact that the aqueous solution viscosity would increase with the addition of salt, which results in difficult mass transfer and also can cause abatement the interaction of analytes with sorbent surface and ultimately reduces the extraction efficiency. Consequently, absence of salt was more suitable for recommended MSPE procedure and all the experiments were accomplished without salt addition.

### 3.3. Reusability of the sorbent

The magnetic nanoscale sorbent can disperse in the plasma medium, and in the presence of magnetic field it can be separated easily. After the extraction of analytes, the sorbent could be conveniently regenerated by rinsing the MNPs sequentially with 2.0 mL of ACN and 2.0 mL double-distilled water during sonication time of 4 min. After drying, the sorbent was applied for subsequent MSPE. The extraction recovery of analytes decreased approximately 10% after 14 cycles of MSPE procedure which indicated a slight loss of the sorption capacity.

### 3.4. Method validation

#### 3.4.1. Analytical performance

To assess the practicability of the developed protocol, the figures of merits including linear dynamic range (LDR), correlation coefficient (*r*^2^), limit of detection (LOD), limit of quantification (LOQ) and extraction recovery (ER) for MET, CANA and EMPA were evaluated under the optimized experimental conditions. Calibration curves were plotted using different spiked human plasma with three independent measurements for each point. The enrichment factor (EF) was defined from the ratio of slope of preconcentrated samples to those obtained without preconcentration method. The extraction recovery (ER) was calculated by the following equation:

1 ER% = EF × (*V*_Final volume_/*V*_Initial volume of plasma_) × 100.

**Fig. 6:**
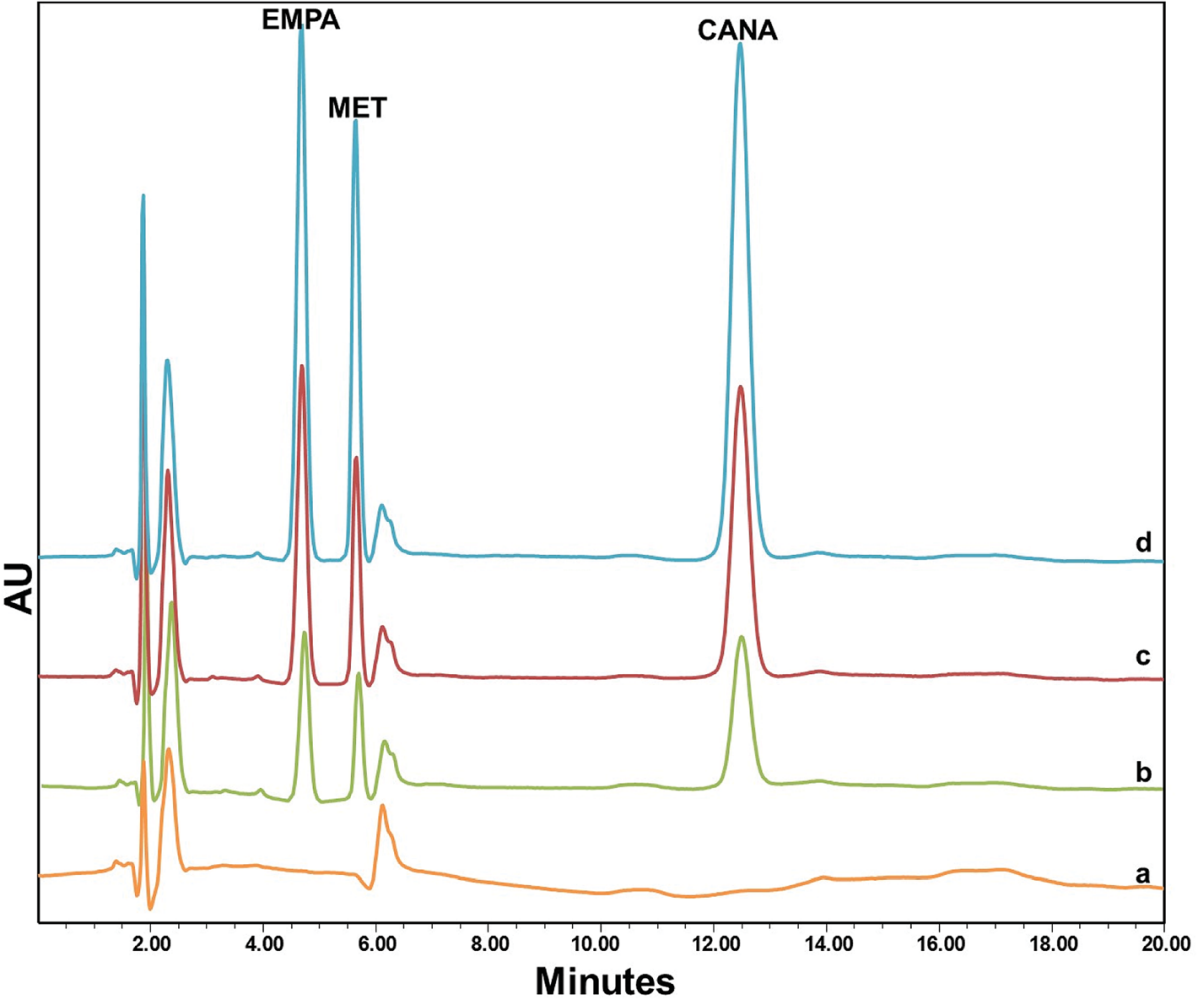
The chromatograms of EMPA, MET and CANA in human plasma; (a) blank; spiked plasma at (b) 100.0 ng mL^−1^, (c) 250.0 ng mL^−1^ and (d) 400.0 ng mL^−1^ concentration levels of each drug.

The analytical performance data are summarized in Table 1. The calibration curves were linear over the concentration ranges of 5.0–1200.0 ng mL^−1^ for EMPA, 20.0–1800.0 ng mL^−1^ for MET and 5.0–1000.0 ng mL^−1^ for CANA while correlation coefficient varied from 0.993 to 0.997. The LODs based on S/N = 3 were 1.3 ng mL^−1^, 6.0 ng mL^−1^ and 0.8 ng mL^−1^ for EMPA, MET and CANA, respectively. The chromatograms for blank and spiked human plasma are exhibited in Fig. 6 and indicated that there was no remarkable interference in the whole analytical protocol.

**Table 1:** Analytical characteristics of the proposed MSPE-HPLC-UV^a^

Analyte	LDR (ng mL^−1^)	Linear equation	*r* ^2^	LOD (ng mL^−1^)	LOQ (ng mL^−1^)	EF	ER% (*n* = 3)
EMPA	5.0–1200.0	*Y* = 173*X* + 195	0.996	1.3	5.0	18.4	92.0
MET	20.0–1800.0	*Y* = 105*X* + 76	0.993	6.0	20.0	17.3	86.5
CANA	5.0–1000.0	*Y* = 225*X* + 270	0.997	0.8	5.0	18.5	92.5

a LDR: Linear dynamic range; *r*^2^: correlation coefficient; LOD: limit of detection; LOQ: limit of quantification; EF: enrichment factor; ER: extraction recovery (250.0 ng mL^−1^ of each drug was used).

#### 3.4.2. Precision and accuracy

The intra-day and inter-day precisions and accuracy of the method were evaluated by assaying spiked samples at QC levels (25.0, 500.0 and 1000.0 ng mL^−1^) in the same day and in three consecutive days, respectively. The relative standard deviations (RSDs) of the intra-day and inter-day assays and accuracy values are summarized in Table 2. The RSDs of the intra-daily tests are less than 7.5% and the RSDs of inter-daily tests are less than 8.5%. These results prove the reasonable accuracy and precision of the method for monitoring of antidiabetic drugs in biological matrix.

**Table 2:** Intra-day and inter-day precision and accuracy for quantification of EMPA, MET and CANA in human plasma^a^

Drug	Concentration (ng mL^−1^)	Intra-day, *n* = 9			Inter-day, *n* = 12		
		Found value ± SD (ng mL^−1^)	RSD (%)	Accuracy (%)	Found value ± SD (ng mL^−1^)	RSD (%)	Accuracy (%)
EMPA	25.0	26.8 ± 1.3	4.8	7.2	22.9 ± 1.2	5.2	−8.4
	500.0	529.0 ± 21.7	4.1	5.8	545.5 ± 27.3	5.0	9.1
	1000.0	1036.0 ± 39.4	3.8	3.6	958.0 ± 42.1	4.4	−4.2
MET	25.0	26.6 ± 1.5	5.6	6.4	26.8 ± 1.7	6.3	7.2
	500.0	530.0 ± 38.2	7.2	6.0	543.1 ± 36.9	6.8	8.6
	1000.0	1044.0 ± 42.8	4.1	4.4	1050.0 ± 33.6	3.2	5.0
CANA	25.0	26.5 ± 1.3	4.9	6.0	27.0 ± 2.3	8.5	8.0
	500.0	520.5 ± 39.0	7.5	4.1	526.5 ± 41.1	7.8	5.3
	1000.0	1039.0 ± 41.6	4.0	3.9	1066.0 ± 42.9	4.0	6.6

a RSD (%) = 100 × SD/mean; accuracy (%) = (mean concentration found − known concentration)/(known concentration); intraday (*n* = 12) = triplicate samples within a series of six measurements on different days.

### 3.5. Application of the method to pharmacokinetic assessment

In order to evaluate the capability and validity of the proposed method, it was applied for determination of EMPA, MET and CANA in human plasma and main pharmacokinetic data were calculated. Eight healthy fed male volunteers within the age range of 28–42 years old were enrolled in these studies. Oral administration of a fixed-dose combination Invokamet tablet (50 mg CANA/500 mg MET) was performed to four volunteers and the blood samples were collected at 0, 1, 2, 3, 4, 5, 6, 8, 10, 12 and 24 h after drug administration. The plasma samples were immediately transferred into a clean polypropylene tube and stored at −18 °C until being used. All assessments were approved by “The Committee For Research Ethics” of Department of Pharmacy and Pharmaceutical Sciences Research Center, Tehran University of Medical Sciences. In the next experiments, a single oral dose administration of Jardiance tablet (25 mg EMPA) was performed to four volunteers and the blood samples were collected and treated as the previous experiments. Each real sample was extracted at optimal conditions by the proposed procedure and the mean plasma concentration–time curve was obtained. The main pharmacokinetic parameters including *T*_max_, *C*_max_, AUC_0–*t*_, AUC_0–∞_, and *T*_1/2_ are summarized in Table 3. These data demonstrate the flexibility of the method in multipurpose analytical applications and its validity for trace drug monitoring in complex biological matrix.

**Table 3:** Pharmacokinetic parameters of EMPA, MET and CANA after oral administration of a fixed-dose combination Invokamet tablet (50 mg CANA/500 mg MET) and a single dose of Jardiance tablet (EMPA 25 mg)^a^

Pharmacokinetic parameters	Mean ± SD		
	CANA	MET	EMPA
*T*_max_ (h)	3.2 ± 0.3	4.1 ± 0.5	2.9 ± 0.3
*C*_max_ (ng mL^−1^)	390.4 ± 50.2	859.7 ± 91.2	215.6 ± 44.1
AUC_0–24_ (ng h mL^−1^)	3862.8 ± 426.9	6627.1 ± 699.3	1398.7 ± 122.5
AUC_0–∞_ (ng h mL^−1^)	3973.6 ± 508.5	7022.8 ± 703.6	1426.0 ± 150.8
*T*_1/2_ (h)	12.1 ± 1.4	6.3 ± 0.8	7.2 ± 1.1

a *T*_max_: Time required for reaching maximum plasma concentration, *C*_max_: maximum plasma concentration, AUC_0–24_: area under curve, AUC_0–∞_: area under curve at infinite time, *T*_1/2_ (h): time required for reaching to half concentration.

### 3.6. Comparison with other methods

Comparison of the proposed method with different existing methods for extraction and determination of antidiabetic drugs is provided in Table 4. It was revealed that along with its simplicity, present combination benefits from some advantages such as low LODs, wide linearity ranges, high sensitivities and reasonable recoveries of target analytes with an important emphasis on the reusability of the sorbent which is comparable with existing techniques. These advantages confirm applicability of the present extraction/preconcentration procedure for trace monitoring of antidiabetic drugs in complex plasma matrices.

**Table 4:** Comparison of the presented approach with other methods for determination of different antidiabetic drugs in human plasma

Sorbent/extraction method	Drug	LOD (ng mL^−1^)	DLR (ng mL^−1^)	*r* ^2^	RSD (%)	Separation/detection system	Ref.
Sequential HF-LPME^a^	MET	1	5–2500	0.999	<8.4%	HPLC-UV	48
IP-VALLLME^b^	MET	1400	20 000–2000000	0.9988	<10.8	HPLC-UV	49
IPSPE^c^	MET	3	50–2000	≥0.997	<9%	HPLC-UV	50
SPE cartridge	MET	1480	—	≥0.9992	<13.4	HPLC-ESI-MSn^e^	51
SPE^d^ cartridge	CANA	—	10.3–6019	≥0.99	—	LC-MS/MS	52
LLE^f^	EMPA	—	—	0.9997	<6.99	LC-MS/MS	53
MSPE	EMPA	1.3	5.0–1200.0	0.996	≤5.2	HPLC-UV	This work
	MET	6.0	20.0–1800.0	0.993	≤6.8		
	CANA	0.8	5.0–1000.0	0.997	≤8.5		

a Sequential hollow-fiber liquid phase microextraction.

b Ion-pair vortex assisted liquid–liquid microextraction.

c Ion pair solid phase extraction.

d Solid phase extraction.

e High performance liquid chromatography-electrospray ionization multi-stage mass spectrometry.

f Liquid–liquid extraction.

## 4. Conclusion

The aim of present study was to develop and validate a reliable MSPE method based on PIL-coated Fe_3_O_4_@SiO_2_ nanoparticles for simultaneous extraction, preconcentration and determination of antidiabetic drugs from human plasma. Chemical immobilization of PIL on the surface of Fe_3_O_4_@SiO_2_ core–shell through polymeric bonds offers strong adhesion between the IL and MPNs which prevents ionic liquids leakage. Furthermore, the later modification merges the interesting advantages of MNPs with individual characteristics of ILs resulting significant improvements in stability, reusability, mass transfer capacity and diffusion routes of the nanoscale sorbent. The successful pharmacokinetic study with low dose administration of antidiabetic drugs demonstrated that the current enrichment procedure in combination with HPLC-UV is a valid means for screening purposes. According to these criteria, the validated method has notable potential for further toxicodynamic and bioequivalence studies.

## Conflicts of interest

There are no conflicts to declare.

## Supplementary Material

RA-008-C8RA02109K-s001
