# Supplementary material for: Silica-coated magnetic iron oxide functionalized with hydrophobic polymeric ionic liquid: a promising nanoscale sorbent for simultaneous extraction of antidiabetic drugs from human plasma prior to their quantitation by HPLC
Source: RSC Adv. 2018 Aug 29;8(53):30550–61. doi: 10.1039/c8ra02109k (PMC9085437; doi:10.1039/c8ra02109k)
Supplement: RA-008-C8RA02109K-s001 [file RA-008-C8RA02109K-s001.pdf]

Electronic Supplementary Material (ESI) for RSC Advances.
This journal is © The Royal Society of Chemistry 2018

Electronic Supplementary Material

Silica-coated magnetic iron oxide functionalized with hydrophobic polymeric ionic liquid: promising nanoscale sorbent for simultaneous extraction of antidiabetic drugs from human plasma prior to their quantitation by HPLC

Sahar Badragheh[1], Mohsen Zeeb[2*], Mohamad Reza Talei Bavil Olyai[2]

[1]Department of Chemistry, Karaj branch, Islamic Azad University, Karaj, Iran.

[2]Department of Applied Chemistry, Faculty of Science, Islamic Azad University, South Tehran Branch, Tehran, Iran

[*]Correspondence E-mail: zeeb.mohsen@gmail.com; m_zeeb@azad.ac.ir

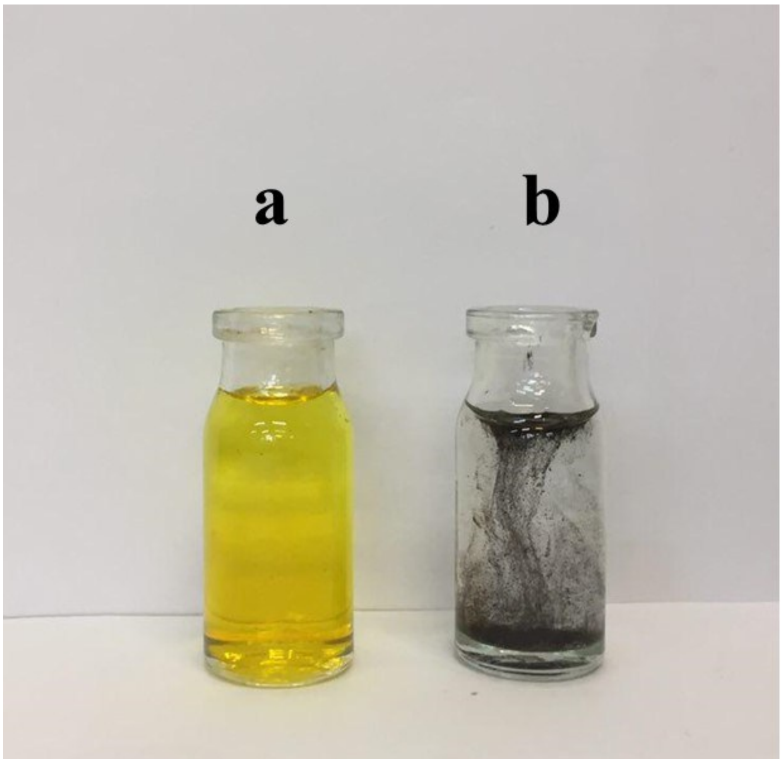

Fig. S1. Comparative acid corrosion experiments for Fe_3O_4 (a) and Fe_3O_4@SiO_2 (b) MNPs.

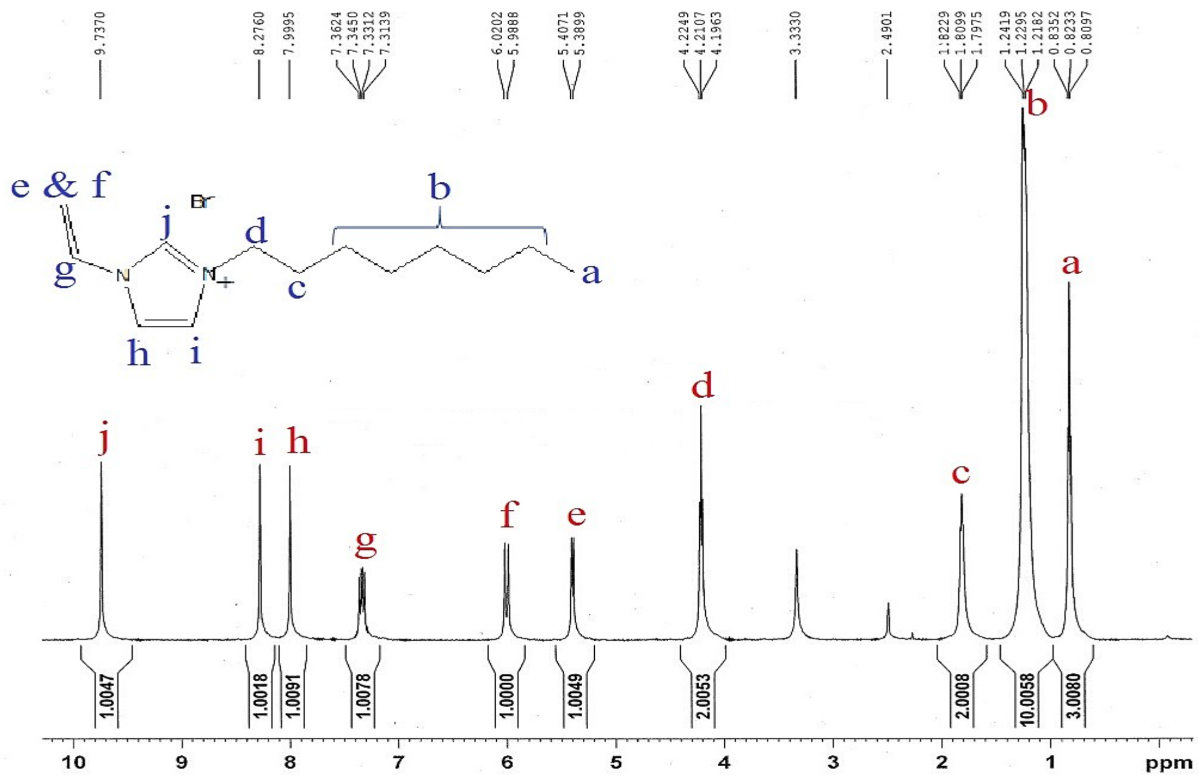

Fig. S2. ^{1}H NMR spectrum of 1-vinyl-3-octylimidazolium bromide monomer.

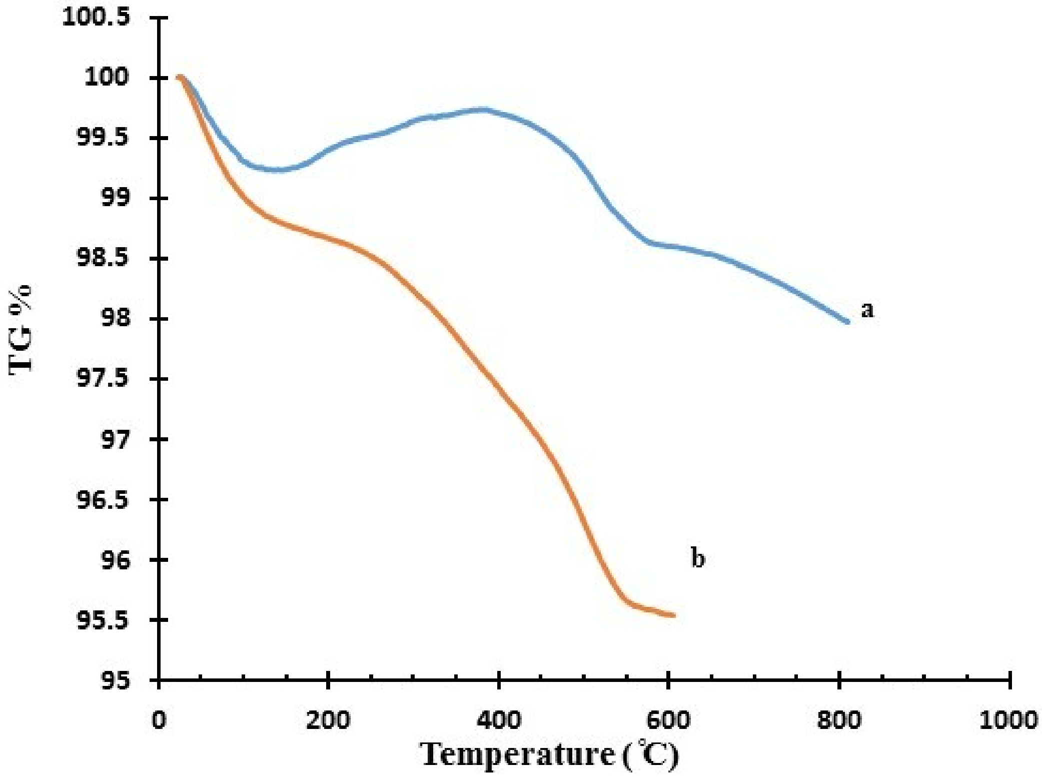

Fig. S3. TGA curves of Fe_3O_4@SiO_2@VTES (a) and Fe_3O_4@SiO_2@PIL-PF_6 (b)

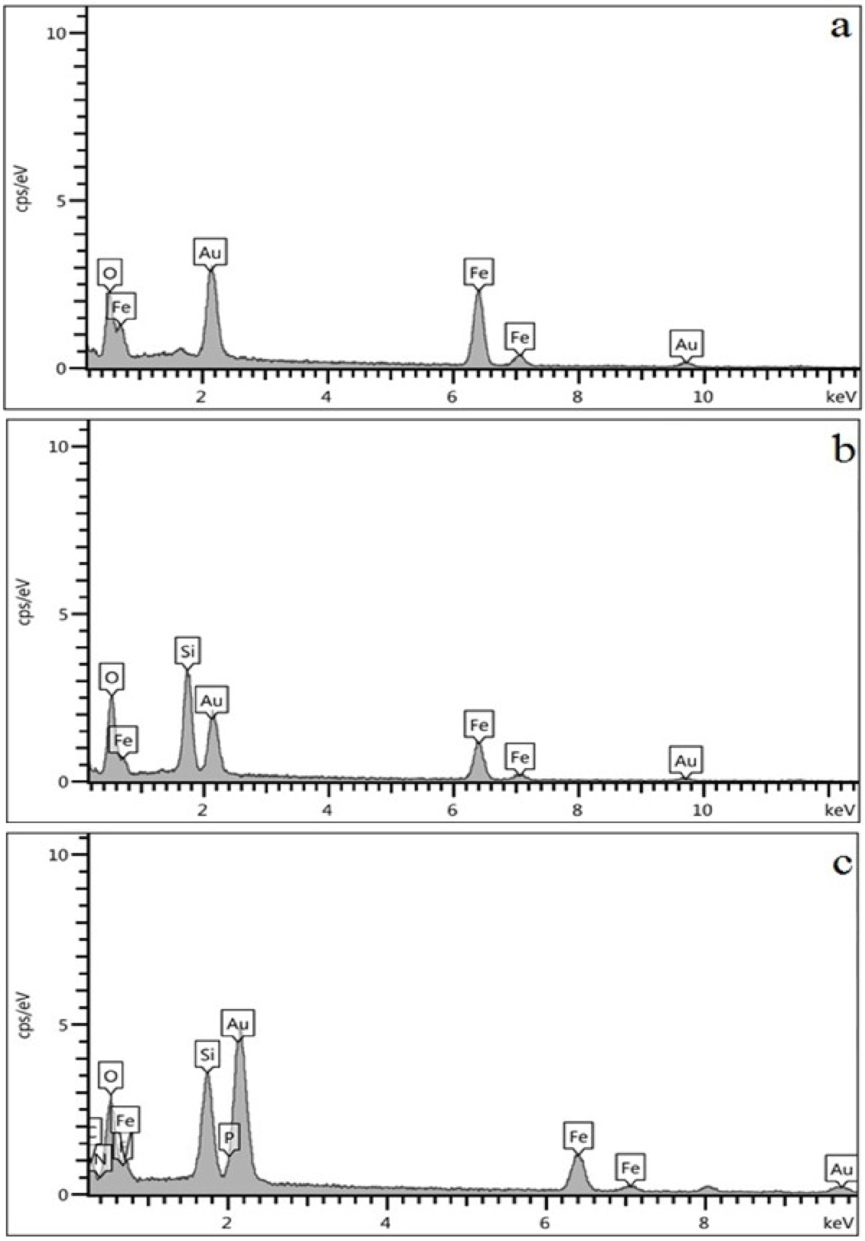

Fig. S4. EDXRF elemental compositions of Fe_3O_4 (a), Fe_3O_4@SiO_2 (b) and Fe_3O_4@SiO_2@PIL-PF_6 (c).

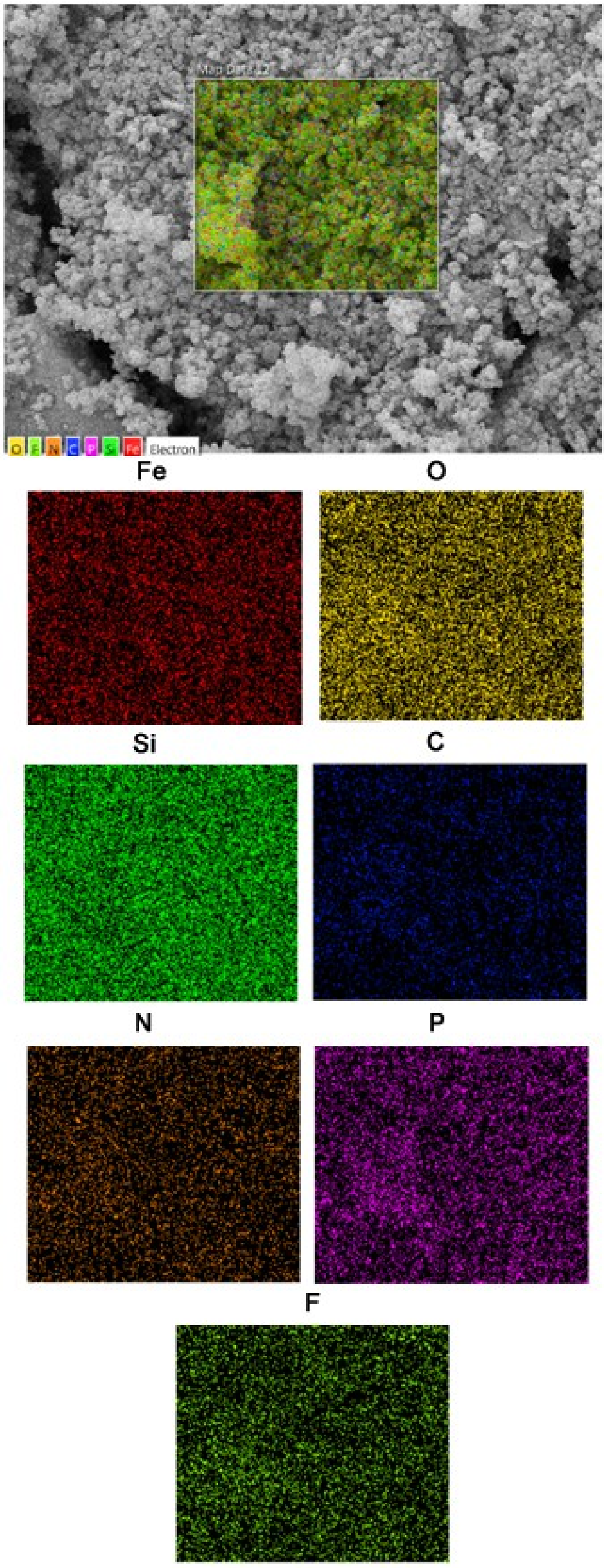

Fig. S5. EDXRF elemental mapping analysis. The top picture shows an overlay of all the elements present in the sample followed by several maps showing the position of each individual element (color figures online).
